# Ecology of the growth of *Anolis nebulosus* (Squamata: Dactyloidae) in a seasonal tropical environment in the Chamela region, Jalisco, Mexico

**DOI:** 10.1002/ece3.4899

**Published:** 2019-01-23

**Authors:** Uriel Hernández‐Salinas, Aurelio Ramírez‐Bautista, Raciel Cruz‐Elizalde, Shai Meiri, Christian Berriozabal‐Islas

**Affiliations:** ^1^ Instituto Politécnico Nacional Centro Interdisciplinario de Investigación para el Desarrollo Integral Regional (CIIDIR) Unidad Durango Durango México; ^2^ Laboratorio de Ecología de Poblaciones, Centro de Investigaciones Biológicas, Instituto de Ciencias Básicas e Ingeniería Universidad Autónoma del Estado de Hidalgo Mineral de La Reforma México; ^3^ School of Zoology Tel Aviv University Tel Aviv Israel; ^4^ Steinhardt Museum for Natural History Tel Aviv University Tel Aviv Israel

**Keywords:** age class, capture–recapture, Chamela Jalisco, growth parameters, sexual maturity, Von Bertalanffy model

## Abstract

Juvenile growth rates are thought to be restricted by available food resources. In animals that grow throughout the year, such as tropical lizards, growth is therefore predicted to be faster during the rainy season. We test this prediction using a population of *Anolis nebulosus*by describing the growth trajectories of both sexes using nonlinear regression models, and we then correlate the growth rates of individuals with food available in the environment, precipitation, and temperature. The Von Bertalanffy model fits the growth rates of the females better, while the logistic‐by‐length model fits the males better. According to both models, the males grew faster than females, reaching slightly smaller sizes at adulthood. Males reached sexual maturity when 35 mm long, at an age of seven months, and females matured at 37 mm (SVL), taking nine months to reach this size. In 1989, juvenile males and females grew more in both seasons (rainy and dry) than adults; for 1990, there were no differences by season or between age classes. These results are interesting since in the 1989 and 1990 rainy seasons, practically the same orders of prey and the greatest abundance of prey available in the environment were registered. A possible explanation could be that predation was more intense in 1990 than in 1989. There is little evidence that food, temperature, and humidity affect growth rates of *A. nebulosus*, refuting our predictions. This is mainly due to the low variation in growth observed in 1990. Therefore we think that the growth of this species reflects a complex combination of ecological and genetic factors.

## 1 INTRODUCTION

Tinkle (1969) predicted that life‐history patterns across lizard species fall along a continuum with two extremes, termed the “fast–slow continuum in life‐history hypothesis” (MacArthur & Wilson, 1967; Pérez‐Mendoza & Zúñiga‐Vega, 2014; Roff, 2002; Schwarz & Meiri, 2017). One end of the continuum comprises small‐bodied species that grow fast, mature early, and lay small (sometimes fixed‐sized) clutches of eggs in quick succession (Clobert, Garland, & Barbault, 1998; Dunham, Miles, & Reznick, 1988; Meiri, Brown, & Sibly, 2012). Individuals of such species often suffer high mortality rates and die young (Scharf et al., 2015). They are also typically oviparous and inhabit tropical environments, conditions in which natural selection favors high reproductive effort over a short time (e.g., multiple clutches during a single reproductive season; Shine & Schwarzkopf, 1992; Roff, 2002). Because the tropics are extremely rich in species, it would also be expected to observe species of long‐lived lizards there (e.g., genera *Iguana*, *Aspidoscelis*).

The other end of the continuum comprises large‐bodied, long‐lived species that suffer low mortality rates. Individuals of these species grow slowly, mature late, and have large clutches (or litters) but reproduce infrequently, usually once per reproductive season, or less often. Such species are typically found in high latitudes where the short seasons restrict the reproductive season and natural selection favors a single large clutch (Roff, 2002; Tinkle, 1969; Tinkle, Wilbur, & Tilley, 1970). Therefore, the growth rate depends on the reproductive strategies developed by the organisms and their ecological and physiological implications of these strategies. The fast growth, and early sexual maturity, have ecological and physiological implications. Species that reach sexual maturity early have more opportunities to produce eggs during the reproductive season, but they may be more likely to be predated upon (Pincheira‐Donoso & Hunt, 2015; Tinkle, 1969). Many studies have supported these hypotheses, revealing great variation in growth patterns between lizard species from tropical (Dmitriew, 2011) and temperate environments (Meiri et al., 2013; Pérez‐Mendoza & Zúñiga‐Vega, 2014). Variation of this type is evident in such life‐history traits as age and size at sexual maturity, fecundity (Meiri et al., 2012; Wang, Zhao, Yu, & Liu, 2011), and survival (Ogutu & Owen‐Smith, 2006; Pérez‐Mendoza & Zúñiga‐Vega, 2014; Scharf et al., 2015).

Lizard growth rate has been studied under two main theoretical approaches: ecological and phylogenetic (Zamora‐Abrego, Zúñiga‐Vega, & Ortega‐León, 2012). The ecological hypothesis interprets growth rate as an expression of food availability, environmental factors (temperature and precipitation), parasite loads, and foraging success (Bronikowski, 2000; Kratochvíl & Frynta, 2003; Pérez‐Mendoza & Zúñiga‐Vega, 2014). In tropical environments, resource abundance is seasonally high, the breeding season (Stearns, 1992; Tinkle, 1969) is long, and predation pressure is more intense than in temperate environments (Ferguson & Brockman, 1980). Selection there favors a “*bet‐hedging*” strategy (Nevoux, Forcada, Barbraud, Croxall, & Weimerskirch, 2010), such that the risk of predation is spread over multiple small clutches (Pincheira‐Donoso & Hunt, 2015). The phylogenetic hypothesis assumes that life‐history is relatively fixed and that phylogeny determines, to a large extent, the trajectory of growth and its relationship to other life‐history characteristics in each species (Caley & Schwarzkopf, 2004; Dmitriew, 2011). If phylogenetic conservatism is rife, closely related species and populations within species are expected to share similar life histories inherited from their common ancestor (Mesquita, Gomes Faria, Rinaldi Colli, Vitt, & Pianka, 2015). At high elevations, growth rate is lower and individuals often reach sexual maturity later and at a smaller body size than conspecific populations at lower elevations (Lemos‐Espinal & Ballinger, 1995; Ramírez‐Bautista, Leyte‐Manrique, Marshall, & Smith, 2011). Therefore, species from tropical and temperate environments differ in their life‐history characteristics (Ramírez‐Bautista, Cruz‐Elizalde, Hernández‐Salinas, Lozano, & Grummer, 2017). Species of the genus *Anolis* lay one egg per clutch but do so multiple times during the reproductive season at intervals as short as two weeks (Andrews & Rand, 1974; Cox & Calsbeek, 2009; Kratochvil & Kubicka, 2007; Meiri et al., 2012). Most of the 426 currently recognized species within the genus *Anolis* (Uetz, 2018) are small‐bodied and fast‐growing (Andrews, 1979; Dunham, 1978), while a few are larger and grow more slowly (Bonneaud et al., 2016; Cox & Calsbeek, 2009). These two groups exhibit parallel differences in survival rates (lower in the small‐bodied group) and reproduction strategies (less frequent clutches in the large‐bodied group; Stamps & Tanaka, 1981; Dmitriew, 2011). Growth rate is often related to sex. For example, male lizards often grow faster than females, attain sexual maturity earlier, and are able to mate at the end of the reproductive season in which they were born (Pérez‐Mendoza & Zúñiga‐Vega, 2014; Webb, Brook, & Shine, 2003).

In both island and mainland populations of *Anolis*, environmental factors can influence life‐history traits, such as growth rates (Zúñiga‐Vega, Rojas‐González, Lemos‐Espinal, & Pérez‐Trejo, 2005). For example, the availability of food was a primary regulator of growth in island populations of *A. oculatus*and *A. limifrons* (Andrews, 1976). At these sites, animals grew slowly and matured at a larger size than members of nearby mainland populations of the same species, perhaps because of lower food abundance on the islands (Andrews, 1976). More recently, some work suggests that the scarcity of food in an insular environment is a common pattern in lizards as in other vertebrates (Covas, 2012) and is often ascribed to a combination of low interspecific competition and predation pressure and high intraspecific competition on islands (Meiri et al., 2014; Novosolov, Raia, & Meiri, 2013).

Schoener and Schoener (1978) found, that in four mainland *Anolis* species, females grew more slowly than males. They suggested that females fed for a shorter time than males, because several groups of adult males foraged in the same places as the females and were monopolizing the food. In *A. carolinensis* from continental United States, Goodman (2010) found that individuals from northern populations (Tennessee) were larger and exhibited slower growth rates than those from the south (Florida), suggesting that northern individuals are able to store more energy as adults to help them better withstand hibernation in the winter months (Michaud & Echternacht, 1995).

**Figure 1:**
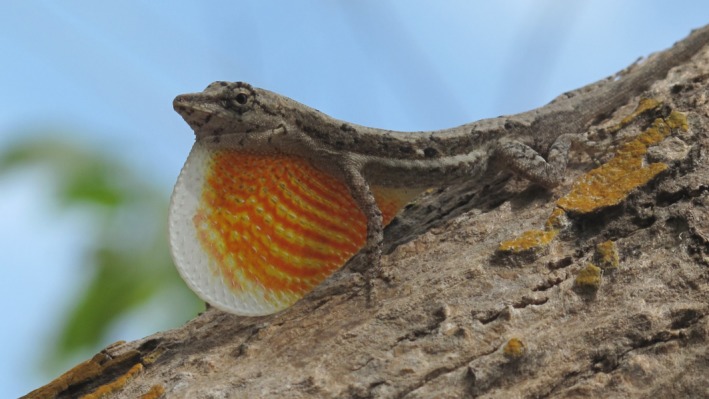
*Anolis nebulosus*(adult male) captured, marked and released at the Estación de Biología Chamela‐UNAM in September 2011. Photograph: Uriel Hernández Salinas

*Anolis nebulosus* (Wiegmann, 1834) is an arboreal lizard species inhabiting tropical dry forests in Mexico (Smith & Taylor, 1950). This is a short‐lived, fast‐growing species with high clutch frequencies and one egg per clutch. Males (Figure 1) and females reproduce in the next reproductive season after birth. Hatching occurs between September and November (rainy season), when food peaks. After reproduction, both females and males die, and the population is maintained by the new generation (i.e., the next cohort; Ramírez‐Bautista, 1995; Ramírez‐Bautista & Vitt, 1997).

If the ecological hypothesis explains variation in growth rates in lizards, such that growth is largely governed by extrinsic environmental factors such as temperature, precipitation, and food availability through the year (Adolph & Porter, 1996; Tinkle, 1969; Zúñiga‐Vega et al., 2005), we predicted that *A. nebulosus* would grow faster in the productive wet season, and faster overall in wetter years in the tropical seasonal environment of the Mexican Pacific. Based on the assumptions of the ecological hypothesis that states that the variation in growth rates is due to environmental factors, our goals are to answer the following questions: (a) Are there differences in growth rates between sexes? (b) Do growth rates vary among age classes, between dry and wet seasons, and between years? (c) What extrinsic factors (e.g., environmental temperature, precipitation, and food availability) promote variation in growth rate of different age classes and sexes between seasons and years?

## 2 MATERIALS AND METHODS

### 2.1 Study area and field work

This study was carried out at the Estación de Biología Chamela (EBCH), Jalisco, Mexico (19°31′N, 105°04′W, Datum WGS 84), specifically around the road that is within the station. This road is 1,200 m long and runs from the main gate of the station to the station facilities. The station is ~7 km from the Pacific Ocean coast at elevations ranging from 55 to 95 m asl. The vegetation type is tropical dry forest, with patches of deciduous forest and desert scrub (Trejo‐Vázquez, 1988). Sampling was conducted from June 1988 to December 1991 for 10 days each month (Ramírez‐Bautista, 1995). However, most individuals were captured, marked, and recaptured during 1989 and 1990 and the growth analyses are therefore derived from 24 sampling trips, January–December 1989 and 1990. Lizards were encountered in trees located along a 1,200 m × 10 m transect; 702 lizards (300 males, 402 females) were captured and marked by toe‐clipping. Toe‐clipping has historically been the most commonly used method for following cohorts within lizard populations over time (Dunham, 1978; Tinkle, 1969; Tinkle et al., 1970). More recently, Guimaraes et al. (2014) and Olivera‐Tlahuel et al. (2017) have pointed out that this method affects the behavior and health of individuals in at least some lizard species and may also affect survival. However, during our study we found no detectable effect of toe‐clipping on the behavior or survival of marked individuals, as reflected in the numbers of captured and recaptured lizards (see results; Ramírez‐Bautista & Vitt, 1997). We agree that for future studies it is necessary to develop more benign methods that are likely to cause less stress to marked individuals. We recorded a total of 1,568 capture–recapture events, with lizards being recaptured between once and 12 times (Ramírez‐Bautista, 1995; Ramírez‐Bautista & Vitt, 1997). For each capture, snout–vent length (SVL) was measured using calipers (±0.01 mm), and weight was measured using a 10 g Pesola scale (±0.1 g). Sex was assessed based on the presence or absence of a conspicuous dewlap (present only in males; Ramírez‐Bautista, 1995). After the data were recorded, the lizards were returned and released at their initial capture location. Sampling took place between 08:00 and 19:00 hr, which was within the daily activity period of *A. nebulosus* (Ramírez‐Bautista, 1995; Ramírez‐Bautista & Vitt, 1997). Field work was authorized by the internal regulations of the Estación de Biología Chamela, UNAM. No individual was sacrificed.

### 2.2 Growth model

Marked lizards comprised two age classes: juveniles (males = 25.0–31.0 mm, females = 25.0–34.0 mm) and adults (males > 32.0 mm, females > 35.0 mm; Ramírez‐Bautista & Vitt, 1997). The determination of the different age classes comes from the reproductive review of this species (Ramírez‐Bautista & Vitt, 1997). Sample size varied across years, age classes, and sexes as follows: 252 adult males (1989: *n* = 75 individuals; 1990: *n* = 177), 92 adult females (1989: *n* = 36; 1990: *n* = 56), 48 juvenile males (1989: *n* = 16; 1990: *n* = 32), and 310 juvenile females (1989: *n* = 54; 1990: *n* = 256; Ramírez‐Bautista, 1995).

Growth rate for recaptured individuals in different age classes (juveniles and adults) was described with the equation GR = (SVL_2_ – SVL_1_)/no. of days, where growth rate (GR) is the difference between SVL of the last recapture event (SVL_2_) and the first one (SVL_1_), divided by the number of days elapsed between them (Dunham, 1978; Zamora‐Abrego et al., 2012). Growth rate was recorded over recapture intervals of 30 to 100 days (the longest period between captures of the same individual). Growth data, assessed by age class, sex, and year, were then fitted to three nonlinear regression models: the Von Bertalanffy model, the logistic‐by‐length model, and the logistic‐by‐weight model (Dunham, 1978; Schoener & Schoener, 1978).

The Von Bertalanffy model assumes that smaller individuals grow faster than larger ones (Dunham, 1978; Zamora‐Abrego et al., 2012; Table 1). The logistic‐by‐length and logistic‐by‐weight models predict that small individuals will grow quickly until they reach an intermediate size, when their growth rates will decrease nonlinearly (Dunham, 1978; Schoener & Schoener, 1978; Zamora‐Abrego et al., 2012; Table 1). Importantly, maximum growth rate is reached at an earlier age in the logistic‐by‐length model than in the logistic‐by‐weight model (Dunham, 1978). The details of the description of each model are reviewed in Dunham (1978) and Schoener and Schoener (1978). The model selected was the one that best evaluated the growth rate for age class, sex, and year (together) and was the one that yielded the lowest mean square error residuals (MSR) and the highest coefficient of determination or correlation (*R* ^2^; Dunham, 1978; Schoener & Schoener, 1978). Once the best growth model was identified, the corresponding differential equation was used to estimate asymptotic body size (*A*_1_) and the characteristic growth parameter (*r*) for each sex (Table 1).

**Table 1:** Mathematical description of the models evaluated in this paper

Model			
	Von Bertalanffy	Logistic‐by‐Length	Logistic‐by‐Weight
Differential equation	GR = *A*_1_ *r* [1 − (*L*/*A*_1_)]	GR = *Lr*[1 − (*L*/*A*_1_)]	GR = (*rL*/3) [1 − (*L* ^3^/*A*_1_^3^)]
Solution	*L* = *A*_1_ (1−*be* ^−^ *^rt^*)	*L* = *A*_1_/(1 + *be* ^−^ *^rt^*)	*L* = [*A*_1_^3^/(1 + *be* ^−^ *^rt^*)]^1/3^
Where	*b* = (1−*L*_0_/*A*_1_)	*b* = (*A*_1_/*L*_0_)−1	*b* = (*A*_1_^3^/*L*_0_^3^)−1

Note

The details of each model can be reviewed in Dunham (1978) and Schoener and Schoener (1978).

*A*_1_: asymptotic snout–vent length; GR: growth rate; *L*: body lengths which correspond to growth rate; *L*_0_: length at hatching; *r*: characteristic growth parameter.

Using the selected models, we estimated growth curves for both males and females using the average SVL of hatchlings (*L*_0_; $\bar{X}$ = 22.1 ± 1.5 mm, range 19.0–24.0 mm, *n* = 11; hatchlings cannot be accurately sexed). With these curves, we were able to estimate the age at sexual maturity for each sex (Dunham, 1978; Schoener & Schoener, 1978; Zamora‐Abrego et al., 2012). We then used ANOVA to identify differences in growth rate between juveniles and adults of each sex, using season (wet and dry) and year (1989, 1990) as the predictors.

To assess the availability of prey in the environment, insects were collected by sweep netting (Southwood, 1978). This method consists of a net attached to a cylindrical aluminum ring 40 cm in diameter attached to a 120 cm long wooden stick. Using this device, we swept 20 strokes on three different sites chosen at random along the transect where the lizards were captured and released. Sweep netting enabled us to catch insects from the air and vegetation, and the arthropods collected were then placed in a plastic jar, and later identified (Southwood, 1978). Insects were sampled two days or one day before finishing field work on each sampling trip. This enabled us to obtain a representative sample of insect abundance in the study area (Ramírez‐Bautista, 1995). Collected insects were euthanized by freezing, preserved in 70% alcohol, and subsequently identified by ARB to the order level. Identifications were corroborated using keys in Triplehorn and Johnson (2005) and other entomological texts. We regarded the different arthropod orders as prey categories. Coleopterans, hymenopterans, and lepidopterans were further categorized as either larvae or adults. We tested whether growth rates (juveniles and adults, males and females together for each year; independent variable) are correlated with temperature, precipitation, the number of prey items, and the number of orders of insects in the environment (numbers of potential prey; dependent variable; Zar, 2014).

Temperature and precipitation data were taken from the EBCH weather station, Universidad Nacional Autónoma de Mexico (UNAM) during fieldwork. Estimates of growth rate were obtained using Statistica 7.0. Means are reported ±1 *SE* unless otherwise indicated. Statistical significance is set to *α* = 0.05 throughout.

## 3 RESULTS

### 3.1 Variation in growth rates between sexes, age classes, and seasons

**Figure 2:**
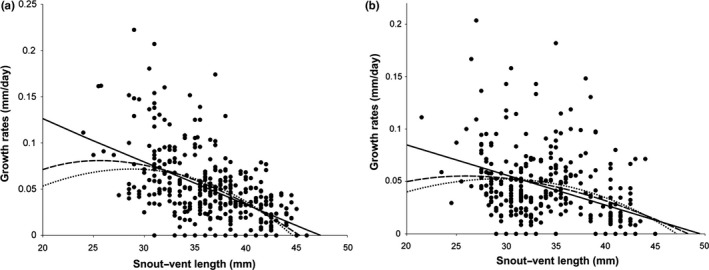
Estimated growth rates as a function of body length for juveniles and adults. Females (a) and males (b) of *Anolis nebulosus* from Chamela, Jalisco, Mexico in 1989 and 1990. Black dots represent growth rate values of individuals. Solid lines represent the Von Bertalanffy models, dashed lines are the logistic‐by‐length models, and dotted lines represent the logistic‐by‐weight model

Observed and estimated body growth rate for females (a) and males (b) for 1989 and 1990 are presented in Figure 2. For males, both the logistic‐by‐length model and the logistic‐by‐weight had practically the same correlation value (*R* ^2^ = 39%); however, the logistic‐by‐length model presented the lowest value of mean square error residuals (MSR 0.3117), so this model provided the best fit for males (Table 2). On the other hand, the Von Bertalanffy model fits the growth data of the females better (*R* ^2^ = 23%; Table 2). Overall, females grew slower (*r* = 0.0017 ± 0.00036) than males (*r* = 0.0053 ± 0.00040; Table 2); however, females reached a slightly larger size (*A*_1_ = 52.8 ± 4.4 mm) than males (*A*_1_ = 46.5 ± 0.9 mm; Figure 2, Table 2).

**Table 2:** Summary of growth parameters for each model in the analysis of growth of *Anolis nebulosus*from Chamela, Jalisco, Mexico

	Model	MSR	*R* ^2^	*A*_1_	*r*
Males (300)	Von Bertalanffy	0.3144	0.3827	50.36 ± 1.9	0.0028 ± 0.00037
	logistic‐by‐length	0.3117	0.3922	46.46 ± 0.9	0.0053 ± 0.00040
	logistic‐by‐weight	0.3123	0.3902	45.07 ± 0.6	0.0076 ± 0.00045
Females (402)	Von Bertalanffy	0.3736	0.2353	52.81 ± 4.4	0.0017 ± 0.00036
	logistic‐by‐length	0.3792	0.2026	47.85 ± 2.1	0.0033 ± 0.00036
	logistic‐by‐weight	0.3832	0.1760	45.81 ± 1.3	0.0051 ± 0.00041

Note

*A*_1_: asymptotic growth parameter; MSR: mean square error residuals; *R* ^2^: coefficient of determination; *r*: characteristic growth parameter.

**Figure 3:**
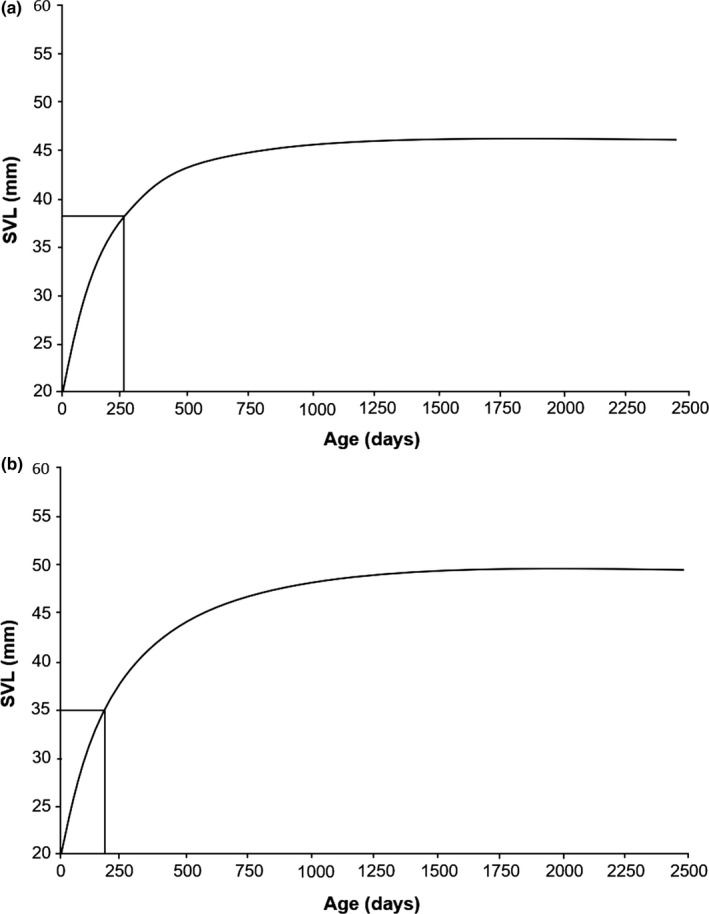
Growth curves for females (a) and males (b) from Chamela, Jalisco, Mexico, estimated with the Von Bertalanffy model (a) and logistic‐by‐length model (b), respectively. Lines indicate the size and age at which the respective sex reaches sexual maturity (combined data; see text)

When estimating a growth curve for each sex, we observed that females reach sexual maturity at 37 mm SVL at an age of 270 days (Figure 3a), while males reached the minimum size at sexual maturity at 35 mm SVL at an age of 210 days (Figure 3b). Considering both age classes (juveniles and adults) of the 702 marked individuals, the analysis of variance revealed that there are no differences in growth rates between years (ANOVA, *F*_2,698_ = 1.274, *p* = 0.2645) or between seasons (ANOVA, *F*_2,698_ = 1.673, *p* = 0.1962), but there is a difference between the sexes (ANOVA, *F*_2,698_ = 11.24, *p* = 0.0008; males: 0.046 ± 0.002, females: 0.039 ± 0.002). Independently in 1989, juvenile males and females grew faster than adults (ANOVA, *F*_2,203_ = 31.22, *p* = 0.0001; Table 3), both in the rainy and dry seasons (ANOVA, *F*_2,203_ = 5.557, *p* = 0.0197; Table 3). In contrast, in 1990 there were no differences between age classes (ANOVA, *F*_2,495_ = 0.027_,_ *p* = 0.8690), season (ANOVA, *F*_2,495_ = 0.003, *p* = 0.9595), or sex (ANOVA, *F*_2,495_ = 0.2.827, *p* = 0.0935; Table 3).

**Table 3:** Average growth rates for each sex and age class of *Anolis nebulosus* by season (dry and wet)

Age class/sex	1989				1990			
	*n*	Dry	*n*	Wet	*n*	Dry	*n*	Wet
Juveniles								
F	42	0.05 ± 0.003	12	0.06 ± 0.012	240	0.03 ± 0.003	16	0.03 ± 0.003
M	10	0.08 ± 0.009	6	0.10 ± 0.016	16	0.05 ± 0.030	16	0.04 ± 0.007
Adult								
F	21	0.02 ± 0.005	15	0.04 ± 0.008	20	0.02 ± 0.006	36	0.05 ± 0.006
M	54	0.03 ± 0.003	21	0.04 ± 0.009	142	0.05 ± 0.002	35	0.04 ± 0.005

Note

The values represent the average and standard error ($\bar{X}$ ± *SE*).

F: females; M: males.

### 3.2 Influence of precipitation, temperature, and prey abundance on growth rate

**Figure 4:**
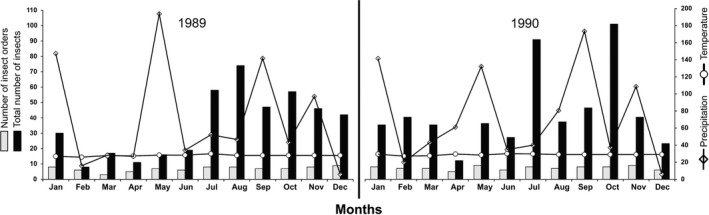
Climate data (lines; precipitation in mm and temperature in °C) and food availability data (bars) for the study site in Chamela, Jalisco, Mexico during 1989 and 1990. The total number of insects refers to the total number of prey items in all categories

For the wet and dry seasons of 1989, 10 prey categories were found; while in 1990, 11 prey categories were found in the wet season and 12 in the dry season (Table 4). For both years, the number of insects was greater for the rainy months (July–November, Figure 4). Temperature was a constant variable throughout both sampling years (Figure 4); precipitation showed high peaks of activity in July–December 1998 and July–November 1999 (Figure 4).

**Table 4:** Categories of prey in the environment collected during sampling years 1989 and 1990 in the region of Chamela, Jalisco, Mexico

Food resource in the environment						
Prey category	1989	Wet season	Dry season	1990	Wet season	Dry season
Acaridae	1		1			
Aranae	161	121	40	167	88	79
Coleoptera (A)	57	49	8	40	29	11
Coleoptera (L)	7	6	1	4	3	1
Diptera	34	31	3	37	29	8
Dermaptera				1		1
Hemiptera	30	25	5	32	25	7
Homoptera	20	14	6	80	66	14
Hymenoptera	53	40	13	61	54	7
Isoptera	1	1				
Lepidoptera (A)	2		2	19	19	
Lepidoptera (L)	11	11		10	8	2
Orthoptera	48	43	5	56	33	23
Psocoptera				1		1
Thysanoptera				3	2	1
Total	425	341	84	511	356	155

Note

Numbers represent number of individuals collected in each prey category.

Adult: A; Larvae: L.

In 1989, there was no relationship between growth rates (of either sex or age class) and the number of prey categories (*r* = 0. 42, *p* = 0.325) or with the number of individual arthropods in the environment (*r* = 0.007, *p* = 0.228) or temperature (*r* = 0.011, *p* = 0.120). However there was a positive relationship with precipitation (*r* = 0.51, *p* = 0.0208). In 1990, there was no relationship between growth rates and the number of prey categories (*r* = 0.001, *p* = 0.335) the number of arthropods in the environment (*r* = 0.001, *p* = 0.348) or temperature (*r = *0.001, *p* = 0.340); however, there was a significant positive relationship between growth rates and precipitation (*r* = 0.18, *p* = 0.006).

## 4 DISCUSSION

In this study, we analyzed the growth rate of juveniles and adults across seasons and years. The potential usefulness of such data is that we test the ecological hypothesis that the growth rates of these individuals are dependent on environmental conditions such as food availability, precipitation, or temperature (Andrews, 1976). The Von Bertalanffy and logistic‐by‐length model equations best described the growth patterns for this population. Such growth patterns are similar to those observed in other *Anolis* species from both island and mainland environments (Andrews, 1982; Goodman, 2010). These populations exhibited an asymptotic growth pattern when they reached sexual maturity, a critical time that demands a large amount of energy be invested in reproduction, which is not only distributed in the production of gonads, but also in the continuous growth process (Andrews, 1982; Cox, Stenquist, & Calsbeek, 2009). We note, however, that while these models were the best among those we tested, their fit to the data was far from perfect (see Figure 2, Table 2).

In 1989, juvenile males and females grew more in both seasons (rainy and dry) than adults; for 1990, there were no differences between seasons or between age classes. This is interesting, since a higher number of prey orders (insects) and greater abundance of prey available in the environment were recorded in 1990 in both the rainy and dry seasons, so we expected to observe different results. In addition, the relationship between growth rates (all 702 individuals together) and precipitation was the only one that was significant for both years, leading us to conclude that in the rainy seasons of 1989 and 1990 there was a high supply of food, and therefore faster growth (Dmitriew, 2011). This suggests that in 1990 there were factors that restricted the growth of the lizards even though environmental conditions were favorable. A possible explanation is that predation may have been more intense in 1990 than in 1989, preventing juveniles and adults from feeding adequately. Another possible explanation is that in 1990 other species of arboreal and large‐sized lizards such as *Sceloporus melanorhinus* or *S. siniferus* (Ramírez‐Bautista, 2004) that live in sympatry with *Anolis nebulosus* monopolized the food (Andrews, 1976, 1979). These are possible causes that could explain the low variation between seasons and sexes for 1990; however, we think that behind this small variation in growth there is a complex mixture of ecological factors that we cannot explain so far.

In terms of food, our results showed that the importance of food for growth rates in both sexes and age classes was contrary to what was anticipated, since the correlations between growth rates and food were not significant. For this reason, we think that it is more important to know the quantity of fat and water (e.g., energy) contained in each type of prey consumed by the lizards than to correlate the number of prey available in the environment with growth rates. For example, in laboratory experiments with *Anolis aeneus*, Stamps and Tanaka (1981) showed that prey containing more water could be positively correlated with growth.

On the other hand, the results also reveal that sexual maturity is attained faster in males, in turn enabling them to increase their reproductive success by mating during their single breeding season, and consequently to increase their fitness (Ramírez‐Bautista, 1995; Ramírez‐Bautista & Vitt, 1997). Another reason could be related to environmental pressures; anole species inhabiting islands grow more slowly than those on the mainland (Andrews, 1979; Goodman, 2010; Hernández‐Salinas, Ramírez‐Bautista, Pavón, & Rosas Pacheco, 2014). Furthermore, the intensity of predation is often stronger on the continent than on islands; thus, lizards in continental environments should grow faster, reaching sexual maturity at smaller sizes as a strategy to offset costs on fitness (Novosolov & Meiri, 2013; Zúñiga‐Vega, Valverde, Rojas‐González, & Lemos‐Espinal, 2007). Therefore, the availability of food and predation intensity are two factors that probably play an important role in the variation of growth rate patterns of males and females in our study population.

Size at sexual maturity and the maximum size reached by males and females in both years were similar to those reported by Siliceo‐Cantero and García (2014). These authors, however, do not report details of growth in different age classes or any effect of season or year. *Anolis nebulosus* hatches at the end of September, and by November–December, males and females had reached the minimum size at sexual maturity; however, they are not actually sexually mature until April–May (males) and June–July of the following year (females) (Ramírez‐Bautista & Vitt, 1997). During this period (end of the wet season), males and females stop growing almost completely (Wang et al., 2011) and devote most of their energy intake to reproduction, survival, movement, and feeding (Ramírez‐Bautista & Vitt, 1997; Woolrich‐Piña, Smith, Lemos‐Espinal, & Ramírez‐Silva, 2015).

Roff and Fairbairn (2007) noted that body growth in vertebrates is connected with other life‐history characteristics, such as age at sexual maturity, number and size of hatchlings, parental investment, and fecundity, among other traits. This pattern reflects a physiological *trade‐off*in which such features of fitness compete for energy (slow growth rate generating low fecundity; Bell, 1980; Angilletta, Steury, & Ears, 2004). In females of *A. nebulosus* as in other lizard species, a slow growth rate could be a *trade‐off*strategy (Warne & Charnov, 2008). For example, Hernández‐Salinas and Ramírez‐Bautista (2015) found that females of this species from an island population were larger and laid larger eggs than their counterparts from the mainland but had lower clutch frequencies. These differences in reproductive characteristics are attributed to different growth patterns, which may be due to a relaxation of predation and scarce agonistic interactions with other island species, in contrast to anoles of the mainland, which may face stronger predation and interspecific competition. Thus, the fast growth rate we found supports the classical predictions of Tinkle (1969): that individuals grow fast to reach sexual maturity at an early age. It also support other ecological hypotheses such as bet‐hedging: that high clutch frequencies enable more eggs to be laid in a reproductive season. Alternatively it may be an adaptation to high predation rates causing accelerated growth rates of young lizards, when predation rate is high (Pincheira‐Donoso & Hunt, 2015).

Dunham (1978) found that variation in growth rate between males and females from the same species and population reflects sexual dimorphism, and usually males attain larger SVL (Andrews, 1982; Zamora‐Abrego et al., 2012). Although males of *A. nebulosus* in our study (both juveniles and adults) showed a higher characteristic growth parameter (*r*) than females (see Table 2), there is no strong evidence that this growth pattern is due to sexual dimorphism (Ramírez‐Bautista & Vitt, 1997, this study). The possible sexual dimorphism present in our study species could be explained by other morphological characteristics, such as the color pattern of the dewlap, which is more colorful (bright orange) and larger in males than in females (Ramírez‐Bautista, 1995) or in head size rather than SVL. These male‐biased characteristics are linked to male–male aggression and agonistic combat between males for access to territory or females (sexual selection; Ramírez‐Bautista, 1995; Scharf & Meiri, 2013).

In females, the slower growth, and later onset of reproductive maturity, are more than compensated for by a longer growth period (almost three months longer than in males). Thus, females reach a slightly greater asymptotic size (*A*_1_). This pattern is related to the fact that between June and November most females of the population have reached sexual maturity. They thus have both vitellogenetic follicles and eggs in their ovaries between July and October (Ramírez‐Bautista & Vitt, 1997). This reproductive effort has such a high cost and after reproducing, the adults (both male and female) die (Dunham, 1978; Ramírez‐Bautista, 1995; Ramírez‐Bautista & Vitt, 1997). Females have an infundibulum (Lozano, Ramírez‐Bautista, & Uribe, 2014; Ramírez‐Bautista, 1995) which stores the sperm of the males to fertilize their eggs long after copulation (Ramírez‐Bautista, 1995; Ramírez‐Bautista & Vitt, 1997). Thus, as was noted, the rapid growth of males can be explained under the assumption that they need to accelerate their growth to establish and defend their territory against invaders by agonistic fighting and/or for access to females for mating (Ramírez‐Bautista, 1995; Ramírez‐Bautista & Vitt, 1997). This demands high energy expenditure and consequently affects their the length of their growth period (April‐May; Ramírez‐Bautista, 1995; Ramírez‐Bautista & Vitt, 1997).

## 5 CONCLUSION

*Anolis nebulosus* grows fast, matures early, and dies young. It lays a single egg per event and produces multiple clutches during the reproductive season. Therefore, the species falls near the fast end of the fast–slow life‐history continuum (Schwarz & Meiri, 2017). All these characteristics are linked to abiotic and biotic factors, such as precipitation, temperature, and food (Ballinger & Congdon, 1980; Meiri et al., 2013; Schwarz & Meiri, 2017). However, our results showed that these environmental factors had little effect on the growth rate of males and females. In this species, males grow faster than females, similar to lizards in other species (Andrews, 1982; Schoener & Schoener, 1978) that reach sexual maturity at an early age and size as the best strategy for increasing or maintaining fitness (Dmitriew, 2011). Although males grow faster than females (*r*), the few differences across seasons and years are not enough evidence to suggest a possible ecological effect in the population analyzed, so our data do not completely support the ecological hypothesis raised at the beginning of this work. For this reason, we cannot claim that the small variation is due to a genetic effect in the species; however, we think that both phylogeny and ecology are important sources of variation in growth rates for many species of lizards (e.g., *Uta stansburiana*, Tinkle, 1967, *Sceloporus occidentalis*, Sinervo, & Adolph, 1989, 1967; *S. merriami*, Grant & Dunham, 1990). Therefore, our results need to be taken with caution because it is necessary to test the growth rate against environmental factors (temperature, precipitation, and food) together with the phylogenetic effect in different regions in the distribution range of *A. nebulosus*.

## CONFLICT OF INTEREST

None declared.

## AUTHORS’ CONTRIBUTIONS

ARB collected data, ARB, UHS and RCE conceived the ideas, designed the study and conducted the analysis of the data. SM and CBI critically reviewed the manuscript for intellectual content. All authors contributed critically to the drafts and gave final approval for publication.

## Data Availability

The data supporting this study and all analyses are available at https://doi.org/10.5061/dryad.cb3tv28.
